# Increasing chitosanase production in *Bacillus cereus* by a novel mutagenesis and screen method

**DOI:** 10.1080/21655979.2020.1869438

**Published:** 2021-01-08

**Authors:** Chaozheng Zhang, Yi Li, Tianshuang Zhang, Hua Zhao

**Affiliations:** Key Laboratory of Ministry of Education Industrial Fermentation Microbiology, Tianjin Key Laboratory of Industrial Microbiology, Tianjin Engineering Research Center of Microbial Metabolism and Fermentation Process Control, College of Biotechnology, Tianjin University of Science and Technology, Tianjin, P. R. China

**Keywords:** *Bacillus cereus*, chitosanase, screening, identification, atmospheric and Room Temperature Plasma Mutagenesis

## Abstract

Chitosan hydrolysis by chitosanase is one of the most effective methods to produce chitosan oligosaccharides. One of the prerequisites of enzyme fermentation production is to select and breed enzyme-producing cells with good performance. So in the process of fermentation production, the low yield of chitosanase cannot meet the current requirement. In this paper, a strain producing chitosanase was screened and identified, and a novel mutagenesis system (Atmospheric and Room Temperature Plasma (ARTP)) was selected to increase the yield of chitosanase. Then, the fermentation medium was optimized to further improve the enzyme activity of the strain. A strain of *Bacillus cereus* capable of producing chitosanase was screened and identified from soil samples. A mutant strain of *B.cereus* was obtained by Atmospheric and Room Temperature Plasma mutagenesis and bioscreening method, and chitosanase activity was 2.49 folds that of the original bacterium. After an optimized fermentation medium, the enzyme activity of the mutant strain was 1.47 folds that of the original bacterium. Combined with all the above optimization experiments, the enzyme activity of mutant strain increased by 3.66 times. The results showed that the Atmospheric and Room Temperature Plasma mutagenesis and bioscreening method could significantly increase the yield of chitosanase in *B.cereus*, and had little effect on the properties of the enzyme. These findings have potential applications in the mutagenesis of other enzyme-producing microorganisms.

## Background

Chitin is the second most abundant biopolymer on the planet after cellulose. Chitin, known as (1,4) −2-acetamide-2-deoxy-β-d-glucan, has molecular weights ranging from hundreds of thousands to millions [1]. Its sources are very wide, common in insects and invertebrate exoskeletons and crustaceans such as fertilizer. However, due to its strong hydrophobicity, chitin is only soluble in certain solvents such as fluoroalcohols and alkaline ice water mixtures [3], and its application scope is greatly limited, and its economic value has not been properly reflected for a long time.

Chitosan, whose scientific name is [1,2] −2-amino-2-deoxy-β-d-glucan, has a relative molecular weight ranging from hundreds of thousands to millions [4]. It is a product of N-deacetylated chitin, which is soluble in most dilute acids. Generally, chitin with a deacetylation degree greater than 50.0% is considered as chitosan. At present, chitosan is mainly extracted from the exoskeleton of shrimp and crab. Chitosan with a degree of polymerization of 2–20 degrees, also known as Chitosan oligosaccharide (COS), is water-soluble and has a variety of biological activities. Therefore, COS has a wide range of potential applications in the fields of medicine, food, agriculture, cosmetics, and so on [2,5]. The COS has attracted more and more attention from laboratory and industry and will become a new functional oligosaccharide. The COS can be obtained by ultrasonic irradiation [6,7], hydrodynamic shearing, and chemical and enzymatic hydrolysis of Chitosan. Enzymatic hydrolysis of Chitosan is one of the best methods for the preparation of Chitosan oligosaccharides [8].

Chitosanases (EC 3.2.1.132) are characterized as enzymes that catalyze the hydrolysis of glycosidic bonds in chitosan to form COS. Chitosanase was first reported from soil microorganisms in 1973 [9] Although chitosanase has been studied for about 50 years [7], only certain microorganisms can produce it. The fungi producing chitosanase are *Aspergillus, Penicillium, Fusarium, Gongronella*, and *Mucor*, and the molecular masses of chitosanases produced are very diverse ranging from 22.5 to 108 kDa [7,10]. Recent studies on chitosanases have been dominated by bacterial species belonging to the genera *Bacillus* [11,12], *Pedobacter* [13], and *Streptomyces*. As COS has good biological activities and great demand, the development of new chitosanases for the preparation of COS is very important. To date, numerous chitosanases with potential industrial applications have been discovered and studied at the biochemical level [6,14–16]. However, these reported chitosanases are all induced enzymes and their production requires chitosan as the inducer [9]. The yields of chitosanases from strains during fermentation are significantly low because chitosan has poor solubility in neutral pH and high viscosity aqueous solutions [17]. Thus, the colloidal chitosan is introduced into the medium as an inducer. The optimization of medium composition and fermentation parameters is an important step in improving the yield of enzymes. However, the application of colloidal chitosan limits the optimization space of medium composition and fermentation parameters. To solve this problem, the use of screening strains that can produce constitutive chitosanase is one of the ways to enhance the yield of chitosanase. Luis et al. reported the production of chitosanase by solid-state fermentation using entomogenic fungi Trichoderma Koningi as the strain, and the optimized yield of chitosanase reached 4.84 U/g (measured by culture base and dry weight) [18]. The aspergillus isolated from fish market waste by Sinha et al. had the highest chitosanase activity (8 U/mL) [19]. A constitutive chitosanase was screened in our laboratory. The enzyme was obtained from *B.cereus* screened from the soil. However, the disadvantage of this strain is that the yield of chitosanase is not high, so it is necessary to increase the yield by mutagenesis.

One of the prerequisites of enzyme fermentation production is to select and breed enzyme-producing cells with good performance. Good enzyme-producing cells should have the following conditions: high enzyme yield; Easy to cultivate management; Stable enzyme production performance; It is beneficial to the separation and purification of enzymes; Safe and reliable. So in the process of fermentation production, the low yield of chitosanase cannot meet the current requirement. Although rational genetic engineering is nowadays the favored method for microbial strain improvement, random mutagenesis is still in many cases the only option. Atmospheric and room-temperature plasma (ARTP) is a newly developed whole-cell mutagenesis tool based on radio-frequency atmospheric-pressure glow discharge plasma which features higher mutation rates than UV radiation or chemical mutagens while maintaining low treatment temperatures [20].

Atmospheric and Room Temperature Plasma (ARTP) is a kind of plasma source of non-equilibrium discharge at atmospheric pressure [21,22]. With ARTP treatment, DNA can be destroyed by high-energy charged particles rather than high temperatures, ultraviolet rays, charged particles, or strong electric fields. ARTP is a new type of physical mutagenesis technology, which has the characteristics of a higher mutation rate, diversity, and at the same time no pollution compared to traditional mutagenesis [22–24]. Therefore, it is more and more widely used in microbial mutation [25–27]. ARTP mutagenesis has been widely used in the screening of yeast and filamentous fungi, and the desired results have been obtained [28,29]. There were some studies that use ARTP mutagenesis to screen Bacillus, which was 1.2–3.3 folds higher than the original strain [30,31].

In this study, a strain of *B.cereus* capable of producing chitosanase was screened and identified from soil samples. The level of chitosanase activity (9.812 U mL^−1^) of *B.cereus* TCCC 10028 is higher compared with *B.cereus* D-11 (4.85 U mL^−1^) [32], *B.cereus* TKU018 (22 mU mL^−1^), and *Aspergillus* sp. CJ22-326 (3.61 U mL^−1^) [33]. Although the chitosanase activity of the strain TCCC 10028 in the broth is lower than that of *Microbacterium* sp. OU01 (118 U mL^−1^) [34] and *Aspergillus* sp. QD-2 (85.816 U mL^−1^) [35], the value suggests that the strain is an efficient producer of constitutive chitosanase. As a production strain of constitutive chitosanase, the biggest advantage is the high yield of enzyme extraction. Whereafter, the ARTP mutagenesis method was used to increase the yield of chitosanase produced by *B.cereus*. The culture conditions of chitosanase production were studied, and the optimum culture conditions were determined, which increased the yield and activity of chitosanase. A new type of chitosanase without chitosan induction was proposed, and its potential application in COS production was studied.

## Materials and methods

### Matrix of the isolation and cultivation media

The matrix of isolation was sampled from a fishpond near the Bohai Sea (Tianjin, China). The enriched medium contained (in g L^−1^) the following: Chitosan powder, 10 g L^−1^; yeast powder, 2.5 g L^−1^; sodium chloride, 5 g L^−1^. The pH was not artificially regulated. Sterilization was executed at 121°C for 20 min.

The seed medium contains peptone 10 g L^−1^, yeast extract 5 g L^−1^, NaCl 5 g L^−1^, and glucose 10 g L^−1^. The pH was adjusted to 6.5. Sterilization was executed at 121°C for 20 min.

The preliminary screening medium consisted of solution A and B. Solution A contained 1% (w/w) colloidal Chitosan and Solution B contained (in g L^−1^) 5 g L^−1^ peptone, 5 g L^−1^ (NH_4_)_2_SO_4_, 1.4 g L^−1^ K_2_HPO_4_, 0.6 g L^−1^ KH_2_PO_4_, 5 g L^−1^ NaCl, 1 g L^−1^ MgSO_4_ · 7H_2_O, and 30 g L^−1^ agar power 30. The pH was adjusted to 6.5. Solutions A and B were sterilized at 115°C for 30 min. Upon cooling to 60°C, Solutions A and B were mixed with a ratio of 1:1. Colloidal Chitosan (1%) was prepared by adding 1 g of Chitosan powder (deacetylation above 90%) in a 250 ml beaker containing 20 ml purified water for 2 min followed by the addition of 30 ml 0.2 mol L^−1^ acetic acid. The solution was constantly stirred until the Chitosan presented as a transparent gel. The pH of colloid Chitosan was adjusted to 5.5 with 1% sodium hydroxide solution and the capacity to 100 ml with distilled water.

The re-screening medium A contained (g L^−1^) 5 g L^−1^ Chitosan powder, 5 g L^−1^ peptone, 5 g L^−1^ (NH_4_)_2_SO_4_, 1.4 g L^−1^ K_2_HPO_4_, 0.6 g L^−1^ KH_2_PO_4_, 5 g L^−1^ NaCl, and 1 g L^−1^ MgSO_4_ · 7H_2_O. The pH of the media was adjusted to 6.5 with 2 mol L^−1^ NaOH and was sterilized at 121°C for 20 min. The re-screening medium B contained glucose instead of Chitosan powder from the re-screening medium A.

Basic fermentation medium contained (g L^−1^) glucose, 5 g L^−1^; yeast extract, 5 g L^−1^; K_2_HPO_4_, 1.4 g L^−1^; KH_2_PO_4_, 0.6 g L^−1^; NaCl, 5 g L^−1^; MgSO_4_ · 7H_2_O, 1 g L^−1^; and Tween-80 1 mL. The pH of the medium was adjusted to 6.0 with 2 mol L^−1^ NaOH and was then sterilized at 121°C for 20 minutes.

### Enrichment and screening of chitosanase-

One gram per liter of the soil sample was used as the inoculum to enrich chitosanase-producing bacteria in a 250 ml conical flask containing 100 ml of the enrichment medium at 30°C for 48 h, with constant shaking at 160 rpm. Using a sterile saline solution, 1 ml bacterial solution in the enrichment medium was successively diluted from 10^−1^ to 10^−6^. Approximately 0.1 ml of each diluent was plated on the preliminary screening medium plate and cultured at 30°C for 3 d. Each sample was done with three replications. Single colonies with good growth and transparent circle were selected for further purification. These colonies were inoculated by a sterile toothpick on preliminary screening medium plates and incubated at 30°C for 3 d. The diameter (d) of the colony and the size of the transparent circle around each colony (D) was measured. The four largest strains on the specific value of D/d were selected for further fermentation screening. The inoculated seed medium was incubated at 30°C for about 8 h, under constant shaking at 160 rpm. The inoculation volume of the seed broth was 2% when the concentration of bacteria in seed broth was 1.0 as determined by OD_600_. The fermentation process was executed at 30°C for 40 h under 160 rpm conditions. Then, crude enzyme liquid was obtained by subjecting the fermentation broth to centrifugation, after which enzyme activity was measured. The strain with the highest enzyme activity was used in the next step.

### Inductivity of chitosanase produced by strains

The strain was inoculated into the re-screening media A and B and cultivated for 40 h at 30°C, with constant agitation at 160 rpm. The inductivity of the enzyme was confirmed based on its activity in different fermentation broths.

### Identification of strain 01

The cellular morphology of strain 01 from the agar plate was observed under the scanning electron microscope (SEM, FEI Apreo HIVac, Czech). Pure culture preserved in a test tube was initially identified by the Biolog test (Biolog ML3420, USA) and by 16S-rDNA sequencing.

### Chitosanase production

Chitosanase production was performed in 250 ml flasks containing 100 ml fermentation medium. According to the 2% (v/v) seed inoculum, the broths were incubated at 30°C, 160 rpm for 30 h in a rotary shaking incubator. After fermentation, the inoculum pellets were separated from the broth by subjecting to a centrifuge at 10,000 × g for 20 min and the supernatant was treated as the crude chitosanase for enzyme activity measurements.

### Detection of chitosanase activity

Chitosanase activity was determined as the rate at which reducing sugar molecules were generated. Enzyme activity was detected by the dinitrosalicylic acid (DNS) method according to a previous study [36] with some modifications. The enzyme activity was calculated by measuring optical density [18] at a wavelength of 540 nm, which was related to the content of reducing sugar in the solution using D-glucosamine as standard [37]. In this experiment, an enzyme activity unit (U) is defined as the amount of enzyme required to produce 1 µmol of glucosamine per minute, 1 μmol/min. The enzyme activity unit per milliliter of fermentation broth (or per milligram of enzyme protein) is U/ml (or U/mg).

### ARTP mutagenesis

The ARTP mutagenesis experiment was slightly adjusted according to the steps in the literature [20]. The bacteria in the logarithmic phase were collected by centrifuging the fermentation broth and washed twice with sterilized saline solution. Then, bacterial suspensions of about 2 × 10^5^ CFU/ml were obtained through diluting them with sterilized saline solution. The slide of Atmospheric and Room Temperature Plasma (ARTP) was coated evenly by 10 µL of suspension and was placed in a hole on the rotary table in the ARTP system; then, the mutagenic procedure was executed. The operation parameters in the mutagenic procedure were as follows: irradiation distance was 2 mm; gas flow rate was 10 L min^−1^; mutagenic power was 100 W; the bacterial strains were treated with 0 s, 10 s, 20 s, 30 s, 40 s, 50 s, 60 s, and 70 s, respectively. Three parallel slides were treated under each mutagenic time. In this experiment, the mortality rate of the bacteria was calculated through a colony count and the mortality curve was plotted. The treated time with a lethality rate of 90% for mutagenesis operation was selected as the operation time of mutagenesis experiments.

### Screening of mutagenic strains

According to the lethality curve, the optimal irradiation time was selected for the experiment, and the slide coated mutant was shaken with hand for 1 min in the 5 mL fermentation medium. Then, the mutated bacterial solution was inoculated in two 100-well microwell plates with 200 μL in each well. The microwell plates were placed in the bioscreen system (OY Growth Curves Biocsreen C, Finland) and cultured for 48 hours. The micropores with high increase in OD_600_ were selected for measuring the activity of chitosanase. And the hole with larger value of enzyme activity/OD_600_ was obtained. The remaining bacterial liquids in the micropores with high enzyme activity were evenly spread on the plate of seed medium and cultured for 24 hours; then, some single colonies were selected and preserved according to the shape and size of colonies on the plate, and then the selected strains were tested for enzymatic activity verification again. The strains with high enzyme activity were obtained by culturing at 30°C for 72 hours. The obtained strains were stored at 4°C.

### Optimization of medium for mutant strain

Because the fermentation medium used by the mutant strain was the fermentation medium of the original strain, the culture medium was optimized. The steepest ascent experiment was used to find the significant factors, and then the response surface methodology was used to determine the best culture medium.

### P-B design for screening significant factors

P-B design was used to screen the factors (medium component) with a significant influence on the production of chitosanase. Seven variables of the fermentation medium producing chitosanase were designed by using the Design-Expert 10 software: glucose (A), yeast extract (B), K_2_HPO_4_ (C), KH_2_PO_4_ (D), Tween-80 (E), MgSO_4_•7H_2_O (F), and NaCl (G). Two levels were tested for each factor as shown in Table 1. The experimental design schemes, including 12 experiments, are shown in Table 2. Three parallel experiments were conducted for each group and the average chitosanase activity for each group was taken as the experimental response.

**Table 1.** Codes and levels of factors for P-B design

Variables	Codes	Levels	
		−1	1
**Glucose (g/L)**	A	4	6
**Yeast extarct (g/L)**	B	4	6
**K_2_HPO_4_ (g/L)**	C	1.2	1.6
**KH_2_PO_4_ (g/L)**	D	0.4	0.8
**Tween-80 (g/L)**	E	1	1.5
**MgSO_4_ (g/L)**	F	0.8	1.2
**NaCl (g/L)**	G	4	6

**Table 2.** P-B design for seven factors with coded values along with the experimental values of chitosanase activity

group	A	B	C	D	E	F	G	Response(U/ml)
**1**	−1	−1	−1	−1	−1	−1	−1	4.1475
**2**	1	1	1	−1	−1	−1	1	4.2525
**3**	1	−1	1	1	1	−1	−1	4.725
**4**	1	1	−1	1	1	1	−1	4.305
**5**	−1	1	1	−1	1	1	1	4.9875
**6**	1	−1	1	1	−1	1	1	4.8825
**7**	1	−1	−1	−1	1	−1	1	3.9375
**8**	−1	1	1	1	−1	−1	−1	5.04
**9**	1	1	−1	−1	−1	1	−1	4.935
**10**	−1	1	−1	1	1	−1	1	4.6575
**11**	−1	−1	1	−1	1	1	−1	4.3575
**12**	−1	−1	−1	1	−1	1	1	5.14

### Steepest ascent design

In the steepest ascent experiment, the effects of variables on the yield of chitosanase were further analyzed and optimized by varying the concentration of significant factors screened by P-B design. The appropriate regions for these significant factors were determined through the steepest ascent experiment. The experimental design and results are shown in Table 3.

**Table 3.** Experimental design and corresponding values of steepest ascent

Steps	Glucose(g/L)	Yeast extarct(g/L)	Tween-80(g/L)	Chitosanaseactivity (U/ml)
**1**	5	5	1	3.9782
**2**	8	9.5	1.1	6.1021
**3**	11	14	1.2	9.6511
**4**	14	18.5	1.3	9.0472

### Response surface methodology (RSM)

B-B design, a type of RSM, was employed to optimize three significant factors (carbon source nitrogen source, and Tween-80) to increase chitosanase production. As shown in Table 4, each factor was investigated at three levels, namely, −1, 0, and +1 (code values) according to previous studies [35,38]. A total of 15 experiments were scheduled (Table 5). The experimental calculation was expressed by the following model equation:‘Y=a_0_ +a_1_X_1_ +a_2_X_2_ +a_3_X_3_ +a_11_X_1_^2^ +a_22_X_2_^2^ +a_33_X_3_^2^ +a_12_X_1_X_2_ +a_13_X_1_X_3_ +a_23_X_2_X_3_’ where X1, X2, and X3 are coded variables; Y is the predicted response; a_0_ is the intercept, a_1_, a_2_, and a_3_ are linear coefficients; a_11_, a_22_, and a_33_ are quadratic coefficients; and a_12_, a_13_, and a_23_ are interactive coefficients. The software Design-Expert 10.0 was employed for the experimental design and data analysis.

**Table 4.** Experimental variables and levels for B-B design

Variables	Codes	Levels		
		−1	0	1
Yeast Extract (g/L)	*X*_1_	11	14	17
Glucose (g/L)	*X*_2_	8	11	14
Tween-80 (g/L)	*X*_3_	0.8	1.2	1.6

**Table 5.** B-B design and results

Run	Independent variables			Experimental results Yield(u/mL)	Predicted results Yield(u/mL)
	X1	X2	X3		
1	−1	0	−1	8.0672	8.1366
2	0	0	0	9.6608	9.5495
3	−1	−1	0	8.7516	8.5455
4	1	−1	0	8.975	8.8754
5	−1	1	0	9.1516	9.0317
6	0	0	0	9.5996	9.5495
7	0	0	0	9.7024	9.5495
8	1	1	0	9.419	9.4056
9	0	−1	−1	8.2358	8.1418
10	0	1	−1	8.6482	8.4509
11	0	1	1	9.0478	8.9306
12	1	0	−1	8.3524	8.1501
13	0	−1	1	8.2358	8.2231
14	1	0	1	9.0566	8.7690
15	−1	0	1	8.0944	8.0787

## Results and discussion

### Isolation of strain producing constructive chitosanase

In the current work, chitosan was used as the sole carbon source to separate strain from the diluents of enrichment culture. Many bacterial colonies and molds were observed in agar plates. A total of 33 colonies with good growth and transparent circles were selected and numbered successively for further purification. To investigate the enzyme production capacity, the strains 01, 06, 10, and 12 with relatively large D/d values were selected for rescreening. Four strains were inoculated into both rescreening media A and B and chitosanase activity was determined after incubation for 36 h. Chitosanase activities in the rescreening medium A for four strains positively correlated with the size of D/d. Strain 01 was the only inoculum found to have chitosanase activity in re-screening medium B broth and therefore considered a consisted enzyme. At present, chitosanase from most microbial sources belongs to the inducible enzyme type and gene expression was mostly controlled by repressor and inducer [4,11,13]. Chitosan was generally used as the inducer and the by-product was the repressor. However, the constitutive chitosanase produced by strain 01 could exactly overcome this problem.

### Identification of strain 01

The colony and cell forms of strain 01 have been shown in Figure 1. The colony was round, with the rough and waxy surface, opaque, white, and looked like frosted glass. Gram staining identified the bacteria as gram-positive. Cells were rod-shaped, with obtuse ends and formed short chains with an approximate size of 0.4–0.6 × 2–3 µm under SEM, as shown in Figure 1.

**Figure 1.**
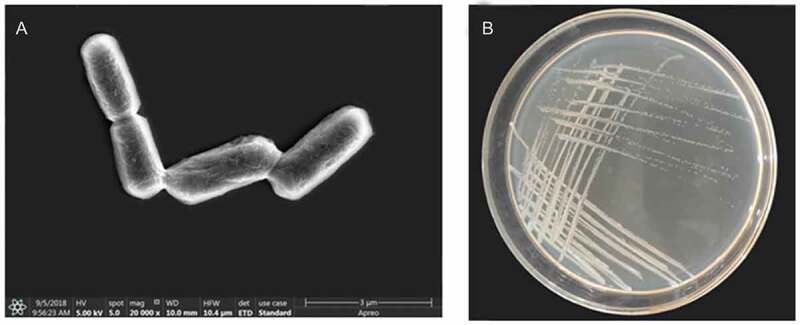
The forms of strain 01(a- cell form, b- colony from)

MicroStation ID System by Biolog (USA) showed that strain 01 can fully utilize 42 of the 95

**Figure 1.**
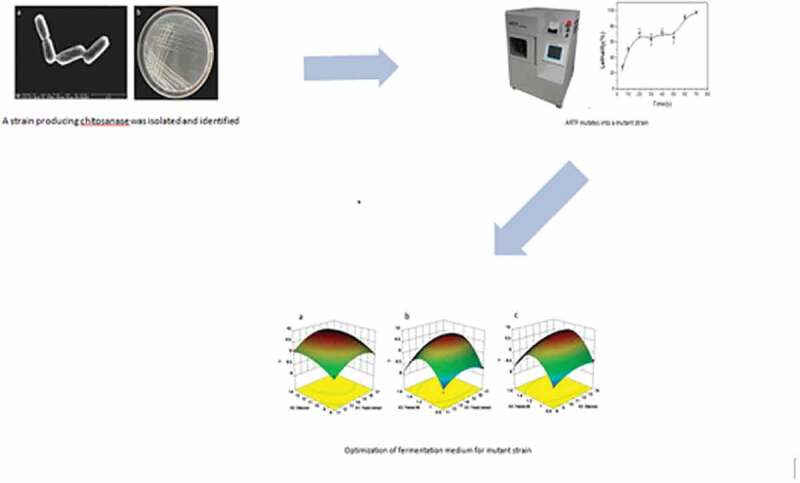
The forms of strain 01(a- cell form, b- colony from)

carbon resources, and it was confidently identified as *B.cereus*. Sequencing with 16S rDNA was blasted into the public database (Gene Bank SUB8731228 SeqID1 MW365346) and revealed that the strain was *B.cereus* at 100% homology. The strain 01 was preserved in Tianjin University of Science and Technology’s Center of Culture Collection (TCCC) and numbered TCCC 10028.

### ARTP mutagenesis and screening of mutant

**Figure 2.**
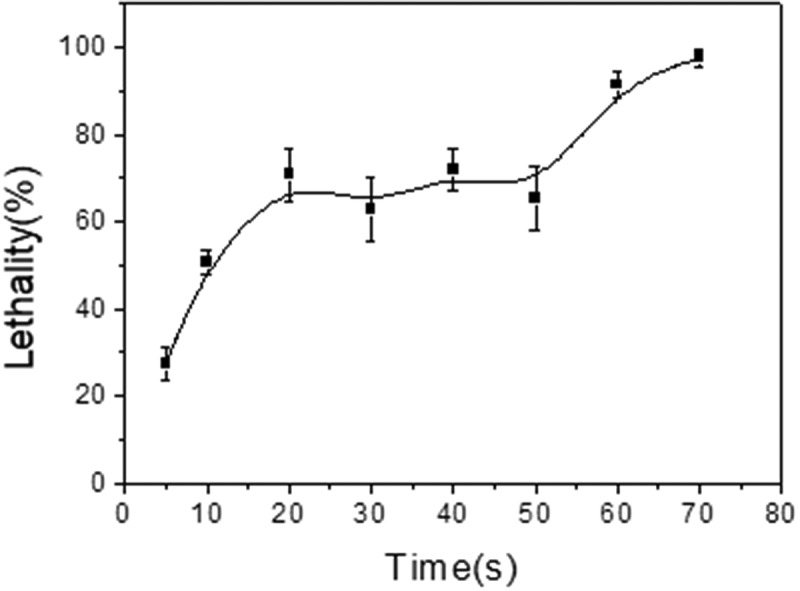
The lethality curve of ARTP mutagenesis

The enzyme activity of the strain TCCC 10028 was only 3.81 U/mL, which could not meet the production requirements. Therefore, ARTP mutagenesis has been used to improve the enzyme activity of the strain. The lethality curve of *B.cereus* mutagenized by ARTP has been shown in Figure 2. When the irradiation time was between 20 s and 50 s, the lethality fluctuated within a constant range (62.7–71.9%). It was inferred that the DNA repair machinery of the bacteria played a positive role, which repaired the damaged DNA in bacteria caused by ARTP. When DNA was severely damaged and/or its synthesis was blocked, the DNA repair machinery was induced and it repaired itself [39]. However, the induction of the repair machinery took time, it was impossible to repair the damaged parts all the time. When the irradiation time was the 60s, the lethality rate of the strain was more than 90% and the DNA repair machinery was no longer working. Then, the 60s was selected for the irradiation time of ARTP. The bacteria after ARTP mutagenesis according to the method were transferred to a bioscreen system. In the case of a 90% fatality rate, only one mutated cell can be assigned to each micropore in bioscreen system. This may reduce the tedious work required for subsequent screening. ARTP has proven to be a reliable and effective microbial breeding system, in which reactive chemical species produced by helium-based atmospheric and room temperature plasma lead to high-frequency random mutations. Please refer to the literature for its detailed working principle [20].

According to the value of OD_600_ obtained from the bioscreen system, 30 wells with a larger increase in OD_600_ value than others were selected, and the enzyme activities were determined according to the after-mentioned method. The yield of chitosanase by the strain was closely related to the concentration of the strain, so several holes with high OD_600_ increase were selected to determine the enzyme activity, and the ratio of enzyme activity to OD_600_ was calculated and arranged in descending order, as shown in Table 6.

**Table 6.** The screening of mutant strains

Well	OD_600_	Activity (U/mL)	Activity/OD_600_	Well	OD_600_	Activity (U/mL)	Activity/OD_600_
202	3.509	3.728676	1.0626	142	3.5	3.320143	0.9486
129	3.515	3.716221	1.0572	196	3.514	3.330107	0.9477
115	3.524	3.591668	1.0192	189	3.506	3.311424	0.9445
221	3.509	3.548074	1.0111	109	3.498	3.105912	0.8879
113	3.505	3.535619	1.0087	182	3.558	3.037408	0.8537
188	3.517	3.492026	0.9929	116	3.523	2.975131	0.8445
126	3.515	3.485798	0.9917	295	3.675	3.093456	0.8418
121	3.538	3.485798	0.9852	284	3.516	2.956448	0.8409
207	3.505	3.435977	0.9803	275	3.599	2.975131	0.8267
131	3.505	3.429749	0.9785	122	3.504	2.881717	0.8224
242	3.557	3.45466	0.9712	233	3.547	2.906627	0.8195
138	3.503	3.392383	0.9684	246	3.531	2.887944	0.8179
276	3.531	3.398611	0.9625	245	3.526	2.869261	0.8137
212	3.5	3.342562	0.955	104	3.503	2.844351	0.812
117	3.505	3.342562	0.9537	193	3.51	2.638838	0.7518

The first six Wells in the ratio of enzyme activity to OD_600_ were selected for the coating to obtain the single colonies. A total of 21 single colonies were obtained from these six holes. The 21 mutants with different colony shapes were isolated and tested enzyme activity in the shaking bottle. The results have been listed in Table 7. The enzyme activity of 15 of the 21 mutant strains was higher than that of the original strain. The highest enzyme activity of the mutant was 9.46 (mutant 202 [1]), 2.49 folds that of the original strain. ARTP mutagenesis has been widely used in the screening of yeast and filamentous fungi, and the desired results have been obtained [28,29]. There were some studies that use ARTP mutagenesis to screen *Bacillus*, which was 1.2–3.3 folds higher than the original strain [30,31]. The yield of mutant strain in this paper reached 2.49 fold that of the original strain, which was consistent with the results reported.

**Table 7.** The mutant strains in shake flask screening

Well	Number	Activity(U/mL)	Well	Number	Activity(U/mL)	Well	Number	Activity(U/mL)
113	(1)	5.2745	129	(1)	3.0228	188	(1)	6.7626
	(2)	6.6940		(2)	4.3640		(2)	6.7528
	(3)	4.3249		(3)	3.3655	221	(1)	4.0997
	(4)	7.2521		(4)	4.1878		(2)	2.1417
	(5)	5.9011	202	(1)	9.4606		(3)	5.0591
	(6)	5.0200		(2)	3.2578			
115	(1)	3.0326		(3)	5.8913			
	(2)	4.2368		(4)	3.2578			

### Stability of enzyme production

In order to investigate the stability of the mutated strain, a continuous transfer experiment was carried out. A mutant displaying high chitosanase activity after the ARTP mutagenic treatments was selected, and its stability of the enzyme production was investigated for six generations by successive inoculations of the strain on chitosanase production medium. The changes in enzyme activity in fermentation broth were very small when the strains were cultured for six generations. By comparing the changes of chitosanase activity in each generation, the stability of enzyme production in the mutant strain was determined.

### Optimization of medium for mutant strain

#### P-B design

Twelve trials were designed with seven variables. The data are listed in Table 2. The chitosanase activities ranged from 3.9375 to 5.13875. Statistical analysis (Table 8) showed that glucose has avery significant effect on the yield of chitosanase (p < 0.01) as well as yeast extract and Tween 80 (0.01 < p < 0.05), whereas other factors have no significant effect (p > 0.05).

**Table 8.** Statistical analysis of PB design

Source	Sum of Squares	df	Mean Square	F Value	p-value Prob > F
**Model**	1.84	7	0.26	25.02	0.0038
**A-Glucose**	1.47	1	1.47	139.72	0.0003
**B-Yeast extarct**	0.017	1	0.017	16.46	0.0154
**C-K_2_HPO_4_**	0.032	1	0.032	2.99	0.1587
**D-KH_2_PO_4_**	0.033	1	0.033	3.11	0.1524
**E-Tween-80**	0.13	1	0.13	12.02	0.0257
**F-MgSO_4_ · 7H_2_O**	7.503 × 10^−3^	1	7.503 × 10^−3^	0.4462	0.4462
**G-NaCl**	1.201 × 10^−3^	1	1.201 × 10^−3^	0.11	0.7526

#### Steepest ascent experiment

According to the regression analysis mentioned above, the steepest ascent test was employed to find the optimal range of three significant variables by increasing concentrations of glucose, yeast extract, and Tween-80. The experimental design and corresponding responses are listed in Table 3. As can be seen, chitosanase production increased with the increasing concentration of significant factors reaching a plateau on the third step. Therefore, these concentrations were chosen for further optimization.

### Optimization of significant variables using RSM

Yeast extract, glucose, and Tween-80 affirmed by P-B design was further optimized using B-B design to further improve the yield of chitosanase. The experimental matrix and corresponding results of the B-B design are shown in Table 4. Multivariate regression analysis was conducted with the statistical software Design-Expert 10.0, and the results are shown in Table 9. The resulting regression equation is obtained using the equation ‘Y = (−7.984) + 1.024 × _1_ + 0.496X_2_ + 10.918X_3_ + 1.222 × 10^−3^X_1_X_2_ + 0.141X_1_X_3_ + 0.083X_2_X_3 –_ 0.041 × _1_^2–^0.024 × _2_^2–^5.606X_3_^2^’ where Y is the predicted value (chitosanase activity), and X_1_, X_2_, and X_3_ are the concentrations of yeast extract, glucose, and Tween-80, respectively.

**Table 9.** Results of regression and variance analysis

Source	Sun of squares	DF	Mean Square	*F*	*P*
Model	4.58	9	0.51	23.40	0.0014
x_1_	0.38	1	0.38	17.36	0.0088
x_2_	0.53	1	0.53	24.59	0.0043
x_3_	0.16	1	0.16	7.35	0.0422
x_1_x_2_	4.840 × 10–4	1	4.840 × 10–4	0.022	0.8872
x_1_x_3_	0.11	1	0.11	5.27	0.0702
x_2_x_3_	0.04	1	0.04	1.84	0.2335
x_1_^2^	0.49	1	0.49	22.57	0.0051
x_2_^2^	0.17	1	0.17	7.87	0.0377
x_3_^2^	2.97	1	2.97	136.59	< 0.0001
Residual	0.11	5	0.022		
Lack of Fit	0.10	3	0.034	12.89	0.0728
Pure Error	5.348 × 10–3	2	2.674 × 10^−3^		
Cor Total	4.69	14			
R^2^	0.9768				
Adjusted R^2^	0.9351				
Predicted R^2^	0.6447				
CV %	1.66				
Adequate Precision	12.513				

In Table 9, the p values of this model and lack of fit were 0.0014 and 0.0728, respectively. This indicates that there is no significant lack of fit in this model and the model, demonstrating the good fit of the model. The F values of the model (23.40) and lack of fit (12.89) also revealed that the experimental data were a good fit with the model.

Results in Table 9 show that linear terms (X_1_, X_2_, and X_3_) and quadric terms (X_1_^2^, X_2_^2^, and X_3_^2^) have significant effects on response values (chitosanase production) (p < 0.05). However, each interaction term has no significant effect on chitosanase production (p > 0.05). The measurement coefficient (R^2^ = 0.9768) indicated that only 2.32% of the total variations cannot be explained by this model. The adjusted coefficient (Adjusted R^2^ = 0.9351) was also quite high, thus verifying the reliability of the model.

**Figure 3.**
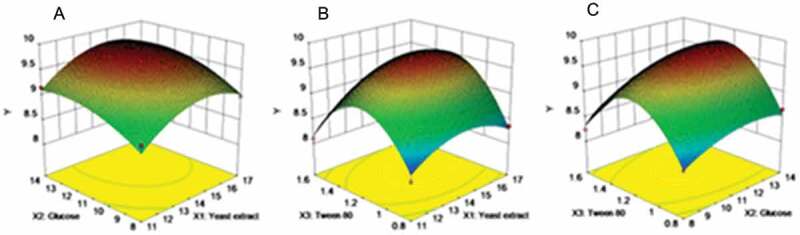
Response surface plot for interaction effects of different factors on chitosanase activity

The response surface diagrams of the combined effects of each independent factor on chitosanase production are shown in Figure 3. When the concentration of Tween-80 was fixed at 1.2 g L^−1^, the response surface plot of the combined effects of yeast extract and glucose on chitosanase production showed that the concentration range of yeast extract and glucose ranged between 15 g L^−1^–16 g L^−1^ and 12 g L^−1^-13 g L^−1^, respectively (Figure 3(a)). The combined effects of yeast extract and Tween-80 on the yield of chitosanase with the fixed initial glucose concentration of 11 g L^−1^ are shown in Figure 3(b). The concentrations of yeast extract and Tween-80 ranged between 15 g L^−1^–16 g L^−1^ and 1.2 g L^−1^–1.4 g L^−1^, respectively. The combined effects of glucose and Tween-80 on the chitosanase production with the fixed initial yeast extract concentration of 14 g L^−1^ are shown in Figure 3(c). The concentrations of glucose and Tween-80 ranged from 12 g L^−1^–13 g L^−1^ and 1.2 g L^−1^–1.4 g L^−1^, respectively. According to the analysis conducted via Design-Expert 10.0, the maximal chitosanase activity of 9.784 U mL^−1^ can be achieved when the concentrations of yeast extract, glucose, and Tween-80 are 15.051, 12.773, and 1.261 g L^−1^, respectively.

To confirm the predicted results of enzyme activity from the Design-Expert 10.0 software, three parallel experiments were conducted on the proposed fermentation conditions. The final chitosanase activity of 9.812 U mL^−1^, which is about 0.28% higher than the predicted value, is obtained under the following conditions: 15.051 g L^−1^ yeast extraction, 12.773 g L^−1^ glucose, 1.4 g L^−1^ K_2_HPO_4_, 0.6 g L^−1^ KH_2_PO_4_, 5 g L^−1^ NaCl, 1 g L^−1^ MgSO_4_•7H_2_O, 1.261 g L^−1^ Tween-80, and initial pH 6.0. This difference may be attributed to the slight changes in experimental conditions. Moreover, initial enzyme activity in the unoptimized fermentation medium was 3.9782 U/mL. Thus, the chitosanase yield had a 1.47-fold increase in the optimal fermentation medium.

The level of chitosanase activity (9.812 U mL^−1^) of *B.cereus* TCCC 10028 is higher compared with *B.cereus* D-11 (4.85 U mL^−1^) [32], *B.cereus* TKU018 (22 mU mL^−1^), and *Aspergillus* sp. CJ22-326 (3.61 U mL^−1^) [33]. Although the chitosanase activity of the strain TCCC 10028 in the broth is lower than that of *Microbacterium* sp. OU01 (118 U mL^−1^) [34] and *Aspergillus* sp. QD-2 (85.816 U mL^−1^) [35], the value suggests that the strain is an efficient producer of constitutive chitosanase. As a production strain of constitutive chitosanase, the biggest advantage is the high yield of enzyme extraction.

## Conclusions

In this study, a chitosanase-producing strain was successfully isolated from marine soil rich in shrimp shells in Tianjin. The results showed that the Atmospheric and Room Temperature Plasma mutagenesis and bioscreening method could significantly increase the yield of chitosanase in *B.cereus*, and had little effect on the properties of the enzyme. By optimizing the fermentation medium, the enzyme activity of the strain was increased 1.37 times, which proved that the optimization of the medium could improve the enzyme activity of the strain. In the final combination with all the above experiments, the enzyme activity of the mutant strain increased by 3.66 times. Although there were many studies on chitosanase production by microorganisms, there have been few reports that ARTP mutagenesis can be used to improve chitosanase production by *Bacillus*. The properties of chitosanase did not change after ARTP mutagenesis. These findings have potential applications in the mutagenesis of other enzyme-producing microorganisms. The further application of this chitosanase laid the foundation.

## Supplementary Material

Supplemental Material

## Research highlights

1. We screened and identified a chitosanase-producing strain from the soil.
2. The activity of chitosanase was improved by ARTP mutagenesis and had little effect on the properties of the enzyme.
3. The enzyme activity of chitosanase was greatly improved by optimizing the fermentation medium of the mutant strain.
4. It has potential application value in mutagenesis of other enzyme-producing microorganisms.
