# Supplementary figures and images for: Increasing chitosanase production in Bacillus cereus by a novel mutagenesis and screen method
Source: Bioengineered. 2021 Jan 8;12(1):266–77. doi: 10.1080/21655979.2020.1869438 (PMC8806256; doi:10.1080/21655979.2020.1869438)

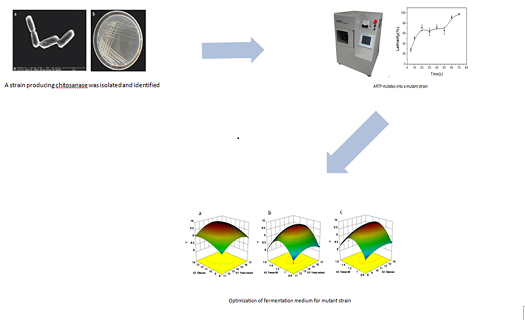

Supplement: Supplemental Material [file KBIE_A_1869438_SM8850.png]
